# Investigating the Properties of Cetyltrimethylammonium Bromide/Hydroxylated Graphene Quantum Dots Thin Film for Potential Optical Detection of Heavy Metal Ions

**DOI:** 10.3390/ma13112591

**Published:** 2020-06-06

**Authors:** Nur Ain Asyiqin Anas, Yap Wing Fen, Nor Azah Yusof, Nur Alia Sheh Omar, Nur Syahira Md Ramdzan, Wan Mohd Ebtisyam Mustaqim Mohd Daniyal

**Affiliations:** 1 Functional Devices Laboratory, Institute of Advanced Technology, Universiti Putra Malaysia, UPM Serdang, Selangor 43400, Malaysia; nurainanas.upm@gmail.com (N.A.A.A.); nuralia.upm@gmail.com (N.A.S.O.); wanmdsyam@gmail.com (W.M.E.M.M.D.); 2 Physics Unit, Centre of Foundation Studies for Agricultural Science, Universiti Putra Malaysia, UPM Serdang, Selangor 43400, Malaysia; 3 Department of Physics, Faculty of Science, Universiti Putra Malaysia, UPM Serdang, Selangor 43400, Malaysia; nursyahira.upm@gmail.com; 4 Department of Chemistry, Faculty of Science, Universiti Putra Malaysia, UPM Serdang, Selangor 43400, Malaysia; azahy@upm.edu.my

**Keywords:** graphene quantum dots, cetyltrimethylammonium bromide, thin film, structural, optical, SPR, heavy metal ions

## Abstract

The modification of graphene quantum dots (GQDs) may drastically enhance their properties, therefore resulting in various related applications. This paper reported the preparation of novel cetyltrimethylammonium bromide/hydroxylated graphene quantum dots (CTAB/HGQDs) thin film using the spin coating technique. The properties of the thin film were then investigated and studied. The functional groups existing in CTAB/HGQDs thin film were confirmed by the Fourier transform infrared (FTIR) spectroscopy, while the atomic force microscope (AFM) displayed a homogenous surface of the thin film with an increase in surface roughness upon modification. Optical characterizations using UV-Vis absorption spectroscopy revealed a high absorption with an optical band gap of 4.162 eV. Additionally, the photoluminescence (PL) spectra illustrated the maximum emission peak of CTAB/HGQDs thin film at a wavelength of 444 nm. The sensing properties of the as-prepared CTAB/HGQDs thin film were studied using a surface plasmon resonance technique towards the detection of several heavy metal ions (HMIs) (Zn^2+^, Ni^2+^, and Fe^3+^). This technique generated significant results and showed that CTAB/HGQDs thin film has great potential for HMIs detection.

## 1. Introduction

In the past few years, there has been astonishing progress in the study and application of various quantum dots (QDs), owing to their versatile chemical and physical properties. QDs is a term that refers to any semiconductor nanoparticles that usually have a size ranging from 2 nm to 10 nm, resulting in quantum confinement and size-dependent optical properties. Comparing QDs with other bulky materials, they possess many special characteristics such as emitting high energy light, high quantum yields, tunable wavelength, and high resistance to degradation [1,2]. The promising and unique properties of QDs provide a tremendous opportunity for QDs to be applied in numerous applications such as imaging, biomedical, drug delivery, sensors, light-emitting diodes, and others [3,4,5,6]. Among all QDs, graphene quantum dots (GQDs) have captivated researchers’ attention for their excellent and special structure-related properties.

When comparing GQDs to other QDs, they have low toxicity, good biocompatibility, are highly soluble in water, optically stable, abundantly available, and can be modified easily due to the functional groups at the edges [7,8,9,10]. There are many oxygen-containing functional groups at the edges of GQDs, thus making them easier to be modified with other materials. This property will in return give researchers many options to modify the GQDs according to the desired application [11,12,13]. This can be achieved through different preparation processes of GQDs. GQDs can be obtained through two classifications of methods, known as “bottom-up” [14,15] and “top-down” [16,17,18]. Both methods will have different starting materials and processes, for example, “bottom-up” requires a simpler process and yields more product compared to the “top-down” method. When using the “bottom-up” approach, the obtained GQDs are more easily modified; however, it very much depends on the target application.

Due to the outstanding properties of GQDs including optical, electrical, structural, mechanical, and thermal, they offer some unique merits for new applications and have been one of the most popular candidates to incorporate with numerous applications [19,20,21,22]. Focusing on enhancing GQDs’ properties, they can be modified, functionalized, immobilized, and doped with other materials and have been proven in many previous studies [23,24,25,26,27,28]. Functionalizing GQDs with the hydroxyl group helps in the reverse of photoionization, improves surface stability, and increases the fluorescent yield-producing hydroxylated GQDs (HGQDs) [25]. A study by Lundstedt et al. proved that the existence of hydroxyl groups also helps in the functionalization of the graphene material [29].

Cetyltrimethylammonium bromide (CTAB), however, is an important positively-charged surfactant that has a long tail of 16-carbon atoms and a head of an ammonium group with three methyl groups attached. CTAB has been successfully employed as a coating agent, stabilizing agent, passivating agent, structure-directing agent in the synthesis of inorganic materials, and also helps in the accumulation of target materials [30,31,32,33,34]. Additionally, CTAB could also enhance the absorption of pollutants which is the reason it is commonly used in wastewater treatment to facilitate the absorption and reaction with the pollutants [35]. Above all the aforementioned favorable properties of CTAB, it is worth noting that the modification of materials with CTAB will enhance the sensing performance of the materials, resulting in improved sensitivity and limit of detection. This is because CTAB improves the hyperchromicity and sensitization to the probe [36]. 

**Figure 1:**
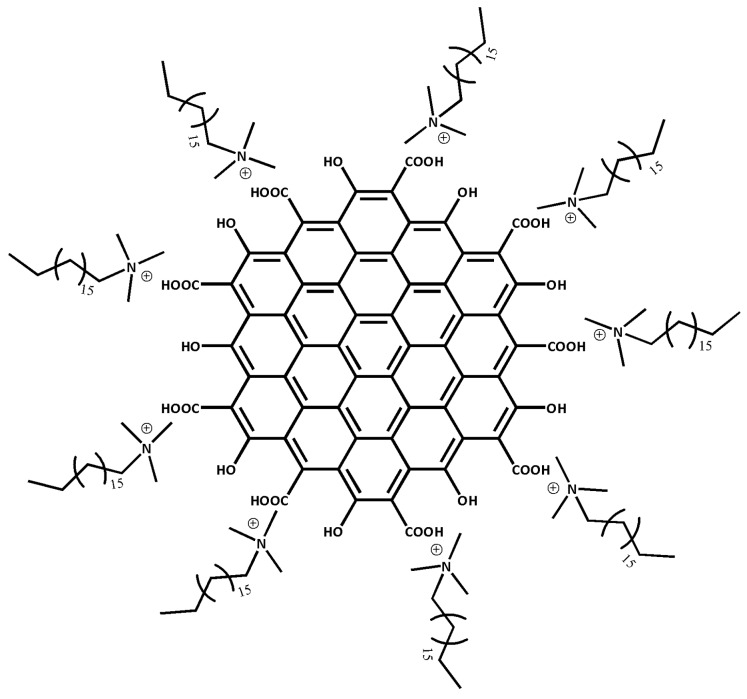
Possible structure of cetyltrimethylammonium bromide/hydroxylated graphene quantum dots (CTAB/HGQDs) composite.

Any metallic chemical element that has a considerably high density and is harmful at low concentrations, can be referred to as heavy metal ions (HMIs). Without realization, long exposure to HMIs may lead to more severe health problems such as organ dysfunction, cancer and breathing problems [37,38]. Due to the awareness of HMIs pollution, there is an urge to produce a high sensitivity material towards the detection of HMIs. Therefore, CTAB is used to modify HGQDs producing cetyltrimethylammonium bromide/hydroxylated graphene quantum dots (CTAB/HGQDs). The CTAB will stabilize the HGQDs surface through the non-covalent interaction with the hydroxyl groups (Figure 1) [39]. There are numerous types of non-covalent interaction such as Van der Waals forces, hydrogen bonding, metal coordination, hydrophobic forces, π-π interaction, and electrostatic forces [40,41]. In this work, the electrostatic force best describes the interaction since it involves the interaction between two charged materials; i.e., positively-charged CTAB and negatively-charged HGQDs [42,43].

This modification is believed to produce a composite material with better sensing properties compared to the individual material. The composite material was then deposited on a gold thin film using the spin coating technique and then applied as a sensing layer using the surface plasmon resonance (SPR) technique. SPR, an optical technique, is widely used because it allows label-free, real-time detection and has a sensitive property in addition to facile preparation of the sample, quick measurement capability, and being cost-effective [44,45,46].

To the best of our knowledge, there is no previous study on the modification of HGQDs with CTAB. Therefore, in this paper, the preparation of CTAB/HGQDs thin film will be described. Then, the properties of the modified composite thin film including structural and optical will be investigated. Additionally, the potential of the thin film as a sensing layer to detect HMIs will also be tested using the SPR technique.

## 2. Experimental Details

### 2.1. Reagent and Materials

The hydroxylated graphene quantum dots (HGQDs) with a concentration of 1 M were purchased from ASC Material, America. Hexadecyltrimethylammonium bromide (CTAB) powder, zinc (II) nitrate (Zn(NO_3_)_2_), nickel (II) nitrate (Ni(NO_3_)_2_), and iron (III) chloride (FeCl_3_) were purchased from Sigma-Aldrich, Steinheim, Germany. Glass coverslips with a dimension of 0.1 mm × 24 mm × 24 mm were supplied by Menzel–Glaser, Braunschweig, Germany. All chemicals used in the experiments were of analytical reagent grade and used without further purification. Deionized water was used throughout the solution preparation.

### 2.2. Preparation of Chemical

Firstly, the HGQDs solution was diluted and reduced to a concentration of 0.5 M using the dilution formula. Next, 18.22 g of CTAB powder was diluted in 100 mL of deionized water to obtain a 0.5 M CTAB solution. The CTAB/HGQDs solution was synthesized according to the previous steps reported by Li et al. (2013), with slight modification where ZnSe QDs solution was replaced with the HGQDs solution [47]. The CTAB solution (0.5 M) was briefly added to the HGQDs solution (0.5 M) with a ratio of 1:4 and sonicated for 30 min to yield a uniform suspension. The HMIs solutions of Zn^2+^, Ni^2+^, and Fe^3+^ with an equal concentration of 0.1 ppm were prepared by dissolving an appropriate amount of individual salts in deionized water [48].

### 2.3. CTAB/HGQDs Thin Film Preparation

Firstly, the acetone was used to clean the bare glass coverslips to ensure that all surfaces were clean and had no fingerprint marks. Then, one side of the glass slip was coated with a gold layer for 67 s to obtain 50 nm thickness using the SC7640 sputter coater machine. Then, approximately 1mL of the as-prepared CTAB/HGQDs solution was slowly deposited on top of the gold layer to fully cover the surface. The glass slip was then spun at 3000 rev/min for 30 s using the spin coating system, P-6708D (Specialty Coating Systems, Indianapolis, IN, USA), to produce a thin layer of CTAB/HGQDs thin film (with a thickness of ~25 nm) on top of the gold layer.

### 2.4. Structural and Optical Instrumentations

The Fourier transform infrared spectroscopy (FTIR) spectrum of CTAB/HGQDs thin film was observed and obtained using the Fourier Transform Infrared Spectrometer model spectrum 100 (PerkinElmer, Waltham, MA, USA), measured within the wavenumber range of 400 to 4000 cm^−1^ at room temperature. The surface morphologies of all thin films were observed and studied through an atomic force microscope (AFM), (Bruker, Biopolis Street, Singapore). Additionally, the AFM analysis will produce information on the roughness of the thin film’s surface. AFM topographic images with scan size 1 × 1 µM were taken at room temperature using the Bruker AFM dimension edge in Scan Asyst Peak Force in tapping mode. The absorbance spectrum was characterized using a UV-Vis spectrophotometer model UV-3600 (Shimadzu, Kyoto, Japan). An LS 55 Photoluminescence Spectrometer (PerkinElmer, Waltham, MA, USA) was used to analyze the photoluminescence (PL) spectra over the wavelength range of 200–800 nm. Both UV-Vis and PL measurements were taken at room temperature.

### 2.5. Surface Plasmon Resonance (SPR) Technique

**Figure 2:**
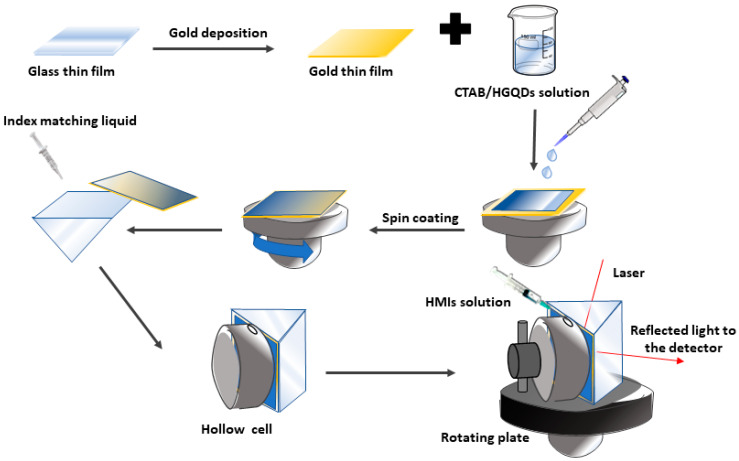
Illustration of the preparation process of the CTAB/HGQDs thin film for surface plasmon resonance (SPR) spectroscopy.

**Figure 3:**
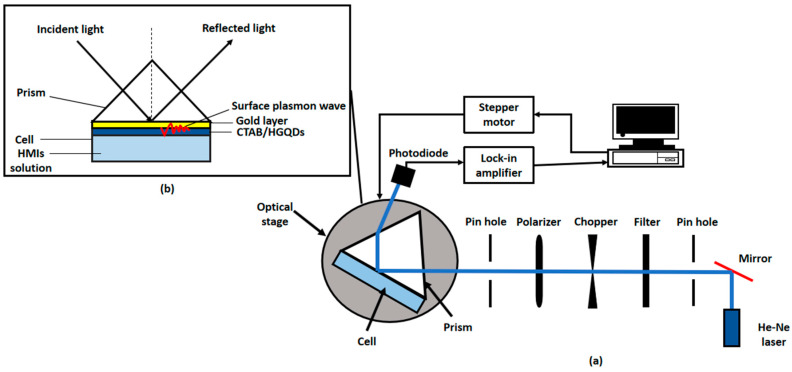
Schematic diagram of (**a**) experimental SPR setup; (**b**) Kretschmann 2D configuration.

A custom-built optical spectroscopy was designed based on the SPR principle to discover the potential of CTAB/HGQDs thin film for sensing Fe^3+^ [45,49,50,51]. In this SPR sensor, a 5 mW, p-polarized He-Ne laser at a wavelength of 632.8 nm will be aimed at the prism (refractive index, n = 1.77861). One side of the prism adhered to CTAB/HGQDs thin film using index matching liquid. The side with CTAB/HGQDs was then attached to a 100 µL cell that holds the HMIs solution. They were mounted on a rotating optical stage guided by a stepper motor MM 3000 (Newport, CA, USA) with a resolution of 0.001° to control the incident light as shown in Figure 2. The incident light passed through the prism and hit the gold layer to generate the surface plasmon waves at the interface. At a specific angle of the incidence light, the SPR response was induced upon the change in the refractive index of the medium in close vicinity of the gold surface [52]. Then, a wide-area photodiode will collect the reflected beam and the SR 530 lock-in-amplifier (Stanford Research Systems, Sunnyvale, CA, USA) will process them. The schematic diagram of the SPR setup based on the Kretschmann configuration is shown in Figure 3.

## 3. Results and Discussion

### 3.1. FTIR Analysis

**Figure 4:**
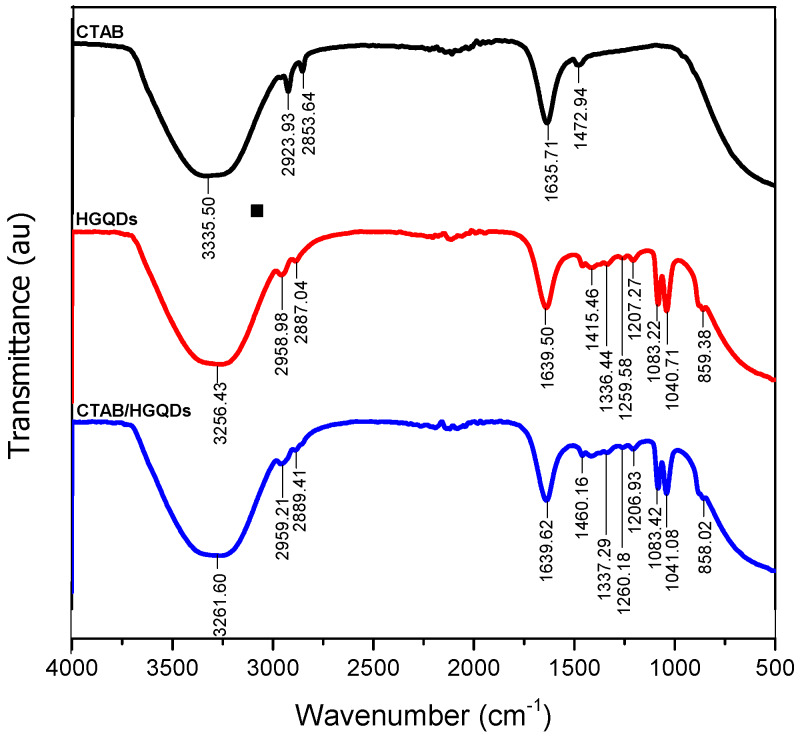
The combined Fourier transform infrared (FTIR) spectrum of the CTAB, HGQDs, and CTAB/HGQDs thin films.

The combined IR spectrum within wavenumbers of 500 cm^−1^ to 4000 cm^−1^ for CTAB, HGQDs, and CTAB/HGQDs thin films are shown in Figure 4. In this work, FTIR spectroscopy was utilized to discover the functional groups existing in each sample and to study the interactions between both composite material CTAB and HGQDs after the modification. 

The IR spectrum of CTAB possesses a strong and broad band at 3335.50 cm^−1^ that can be attributed to the stretching vibrations of the ammonium group in CTAB. Peaks at 2923.93 cm^−1^ and 2853.64 cm^−1^ are attributed to two different C−H band vibrations of the −CH_2_ group in CTAB. The band at 1635.71 cm^−1^ followed by the band at 1472.94 cm^−1^ corresponds to the asymmetric and symmetric stretching vibration of N^+^−CH_3_, respectively. In comparison with other previous reports, similar absorption bands of the FTIR spectrum for CTAB were also observed [53,54,55,56].

As for the HGQDs, the broad absorption band at 3256.43 cm^−1^ arose from the stretching vibration of the O−H bond of the water environment. The following absorptions at 2958.98 cm^−1^ and 2887.04 cm^−1^ are caused by the C−H bonding of the HGQDs. The existence of the stretching frequency at 1639.50 cm^−1^ is a result of the C=C bond, which is the basic component of HGQDs core. The subsequent absorption bands at 1415.46 cm^−1^, 1336.44 cm^−1^, 1259.58 cm^−1^, 1207.27 cm^−1^, 1083.22 cm^−1^, 1040.71 cm^−1^, and 859.38 cm^−1^ have confirmed the existence of carbonyl, carboxyl, and epoxy groups to the edges and onto the basal plane in the HGQDs. The functional groups and the IR spectrum for HGQDs are similar and in agreement with the previous FTIR analysis of GQDs [57,58,59].

On the other hand, the spectrum of the CTAB/HGQDs revealed the combination effect upon the introduction of CTAB to the HGQDs where most peaks shifted to higher wavenumbers when compared to the spectrum of HGQDs [60,61]. It is important to note that the FTIR spectrum of CTAB/HGQDs follows the FTIR spectrum of HGQDs mainly because of the smaller ratio of CTAB compared to HGQDs. There is a strong and broad band at 3261.60 cm^−1^ which corresponds to the stretching vibration of the O−H and N−H groups from HGQDs and CTAB, respectively [53]. The absorption peaks displayed at 2959.21 cm^−1^ and 2889.41 cm^−1^ represent the characteristic CH_2_ stretching vibration bands of CTAB [62,63]. When compared to the HGQDs spectrum, the remaining bands of CTAB/HGQDs have higher wavenumbers at 1460.16 cm^−1^, 1337.29 cm^−1^, 1260.18 cm^−1^, 1206.93 cm^−1^, 1083.42 cm^−1^, 1041.08 cm^−1^, and 858.02 cm^−1^, proving the existence of carbonyl, carboxyl, and epoxy groups, which belong to HGQDs. The results confirmed that the surface modification of HGQDs with CTAB was successful.

### 3.2. Surface Morphology

**Figure 5:**
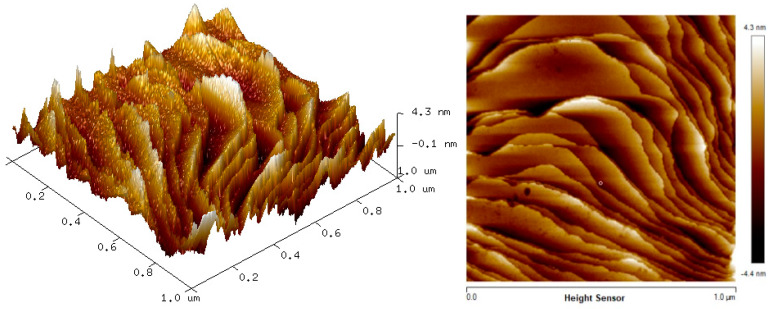
Atomic force microscope (AFM) images of CTAB thin film.

**Figure 6:**
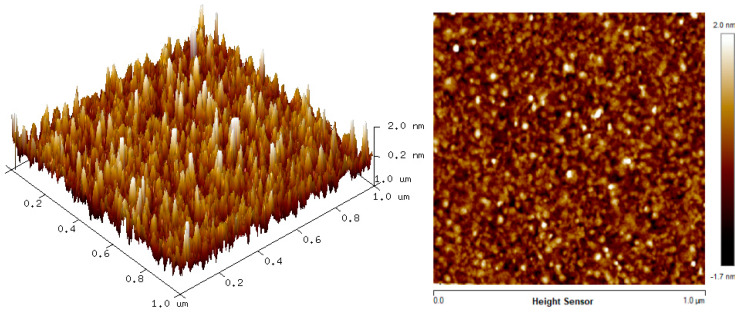
AFM images of HGQDs thin film.

**Figure 7:**
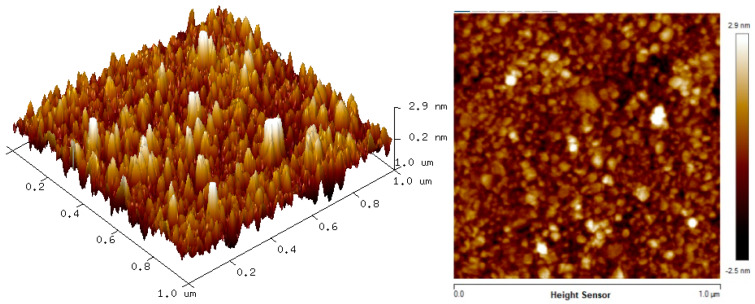
AFM images of CTAB/HGQDs thin film.

The two-dimension (2D) and three-dimension (3D) AFM images of CTAB, HGQDs, and CTAB/HGQDs composite thin films are shown in Figure 5, Figure 6 and Figure 7, respectively. The scan size of 1 µm × 1 µm was chosen in order to get the most precise image. The surface morphologies and the root mean square (RMS) roughness of the thin films when HGQDs are modified with CTAB are then analyzed. It is clear from these images that the surface of all thin films were optimally covered with composite material. The CTAB thin film produces a rough surface with a fish-like scale structure and the RMS roughness obtained for CTAB thin film was 1.22 nm. Figure 6 displays a large number of sharp peaks on top of the HGQDs thin film which look similar to those observed by the previous study [22]. Furthermore, the RMS roughness of the HGQDs thin film increased from 0.546 nm to 0.568 nm upon the modification of HGQDs with CTAB. It can be viewed from the 3D image that the surface of the CTAB/HGQDs thin film showed some granular peaks, likely caused by the addition of CTAB. This is a result of the possible aggregation caused by this process, in agreement with the self-assembly of HGQDs and CTAB [47,64]. 

### 3.3. UV-Vis Absorption

**Figure 8:**
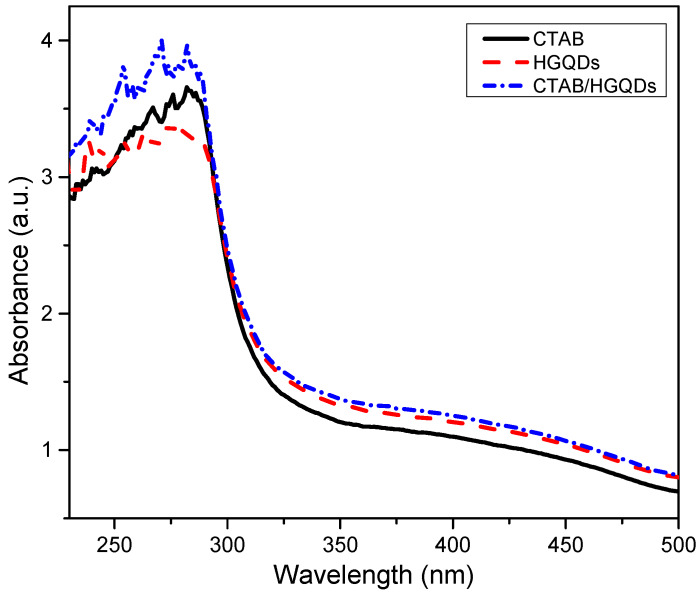
Absorbance spectrum of CTAB, HGQDs, and CTAB/HGQDs thin films.

The UV-Vis absorption analysis of the CTAB/HGQDs thin film was accomplished by observing the absorbance spectrum of the thin film within the wavelength range of 220 nm to 500 nm at room temperature. The absorbance curves for CTAB, HGQDs, and CTAB/HGQDs thin films are presented in Figure 8. The maximum absorption peak for all three thin films can be observed between the wavelengths of 270 nm and 300 nm. CTAB/HGQDs thin film revealed the highest absorbance intensity of 3.98 at the wavelength of 270 nm, whereas HGQDs had the lowest absorbance value of 3.40 at the wavelength of 275 nm. After the modification of HGQDs with CTAB, the intensity of the absorption peak of HGQDs increased, this peak being a result of plasmon resonance in the free electron cloud of carbonaceous material [61]. The absorption peak located at around 270 nm belongs to the n→π* transition of C=O, concurrent with the result in the previous report that also used GQDs-based material [65,66]. At a wavelength of 280 nm, CTAB displayed the absorbance peak with a value of 3.66.

The obtained absorption spectra helped in the calculation of the energy band gap of the thin films. Firstly, the absorbance was determined using the theory relating to Beer–Lambert law where the absorbance of the samples, *A*, related to the ratio of intensities of the emitted radiation in the absence *I_o_* and the presence *I_t_* of the sample.

(1) $$A={log}_{10}\frac{I_{o}}{I_{t}}$$

The transmittance, *T*, of the sample is given by:

(2) $$T=\frac{I_{t}}{I_{o}}$$

Absorbance also depends on the transmittance, thus,

(3) $$A=-{log}_{10}T$$

absorbance coefficient, *α*, is another useful quantity that can be used when measuring samples of differing thickness. The absorbance coefficient is stated as

(4) $$\alpha =2.303\frac{A}{t}$$

In this equation, *t* is the thickness of the sample in units of metre (m), and eventually, the absorbance coefficient, *α*, is to be described in units of m^−1^. Moreover, the values of the energy band gap of all three composites can be measured by replacing the absorbance value and using Tauc relation, where

(5) $$\alpha =\frac{k{\left(hv-E_{g}\right)}^{\frac{1}{2}}}{hv}$$

The rearrangement of the equation will give;

(6) $${\left(\alpha hv\right)}^{2}=k\left(hv-E_{g}\right)$$

**Figure 9:**
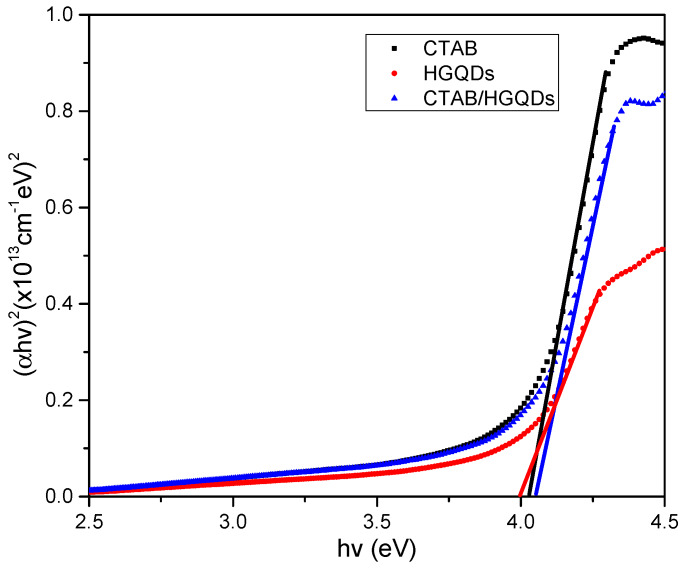
Determination of the optical band gap from (*αhv*)^2^ versus *hv* of all thin films.

Given that *α* is the absorption coefficient, *hv* is the photon energy, *E_g_* is the optical energy band gap, and *k* is constant and substituting *n* = *l*/2 for the direct transition. Then, a graph of (*αhv*)^2^ against *hv* is plotted and the intersection of the straight line on the x-axis is taken as the value of the optical band gap, *E_g_* [67]. The plotted graph of (*αhv*)^2^ versus *hv* for all thin films is shown in Figure 9. The thin film has a direct optical band gap, verified by the linear portion of the plotted graph at the absorption edge. The optical band gap energies, *E_g_*, obtained for CTAB, HGQDs, and CTAB/HGQDs thin films were 4.027 eV, 4.000 eV, and 4.052 eV, respectively, and it is expected that derivatives of higher conductivity show higher absorbance. The variations of the energy band gap value are likely due to the presence of the CTAB solution. By adding CTAB to HGQDs, the optical band gap increased, which is in line with the previous study by Ganesan et al. (2017) [68].

### 3.4. Photoluminescence Emission

**Figure 10:**
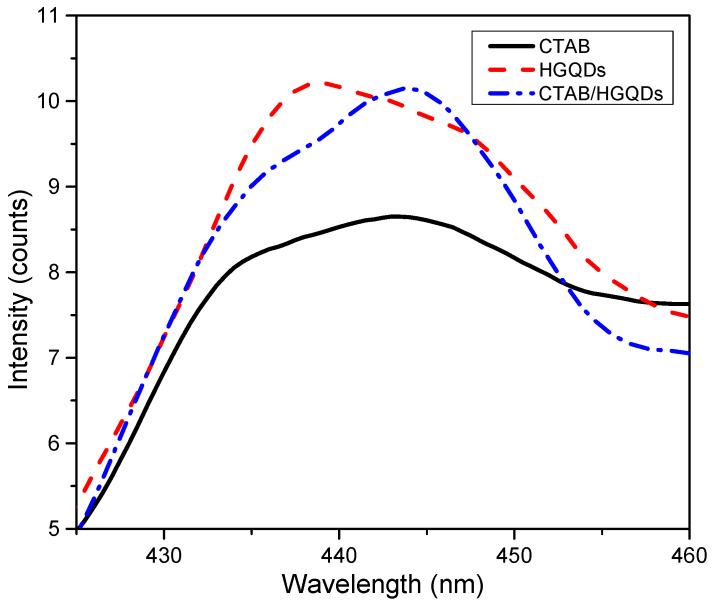
Photoluminescence (PL) spectra of CTAB, HGQDs, and CTAB/HGQDs thin films.

The PL spectra of CTAB, HGQDs, and CTAB/HGQDs thin films under the excitation wavelength of 250 nm at room temperature are presented in Figure 10. The PL spectra of HGQDs thin film showed a maximum emission peak at 438 nm, while both CTAB and CTAB/HGQDs thin films revealed maximum emission peaks at around 444 nm. Comparing both PL spectra of HGQDs and CTAB/HGQDs thin films, the intensity of PL emission for CTAB/HGQDs thin film was slightly lower than HGQDs thin film. When adding CTAB to other materials, its dispersion was enhanced, resulting in an increase in photo-induced charge separation and the lowering of the defect densities [64]. It can be concluded that all three thin films exhibited blue luminescence.

### 3.5. SPR Test of CTAB/HGQDs Thin Film

**Figure 11:**
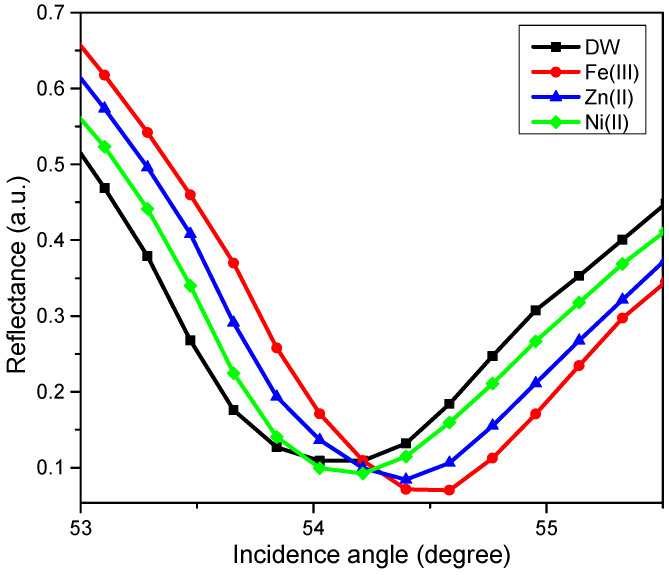
The SPR curves of CTAB/HGQDs thin film in contact with deionized water and 0.1 ppm of Zn^2+^, Ni^2+^, and Fe^3+^ solutions.

The SPR test using novel CTAB/HGQDs thin film began by finding the resonance angle of deionized water to be used as a reference. The resonance angle was found to be 54.10°. Then, the thin film was exposed to the Zn^2+^, Ni^2+^, and Fe^3+^ solution with a concentration of 0.1 ppm. The HMIs solution was injected into the cell in turns and left for a while before measuring the reflectance values. Figure 11 shows the SPR curves of CTAB/HGQDs when in contact with deionized water and the HMIs solution. The SPR curves of all metal ions shifted to the right side of the SPR curve of deionized water due to the increasing refractive index upon the exposure to the HMIs which was consistent with previous studies [69,70]. In summary, SPR worked by observing the changes of the refractive index of the active layer as a result of the interaction of the active layer with the targeted metal ion [71]. The possible interaction mechanism can be attributed to the ketones from HGQDs, which can act as ligands for the metal ions, as expected from the possible structure in Figure 1.

**Figure 12:**
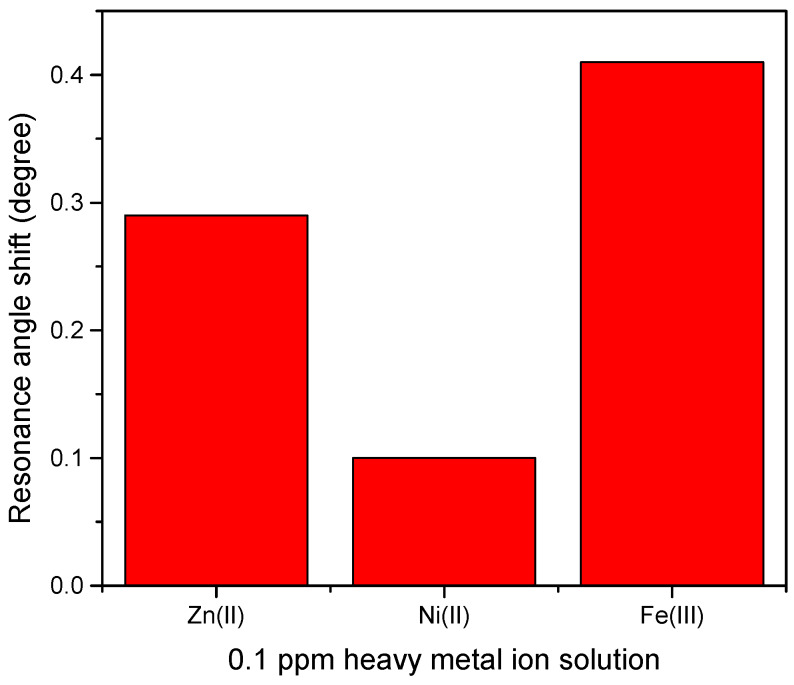
Resonance angle shift of CTAB/HGQDs thin film with different metal ion.

Therefore, by measuring the difference of resonance angle between samples and deionized water as a reference, the shift of resonance angle was determined and plotted for comparison as shown in Figure 12 [72]. Figure 12 demonstrates that the highest shift of resonance angle belongs to Fe^3+^ at 0.41° followed by Zn^2+^ and Ni^2+^ at 0.29° and 0.1°, respectively. The possible explanation of this result is that Fe^3+^ has a greater number of charges than the other ions. However, the other two HMIs that have the same value of charge, Zn^2+^ which is more electronegative than Ni^2+^, may have a stronger interaction with CTAB/HGQDs layer, thus, producing a greater shift of resonance angle [73]. Compared with other materials (Table 1), the as-prepared CTAB/HGQDs in our work have a lower detection limit for the HMIs, which would further improve the practical application when using SPR spectroscopy in the future.

**Figure 13:**
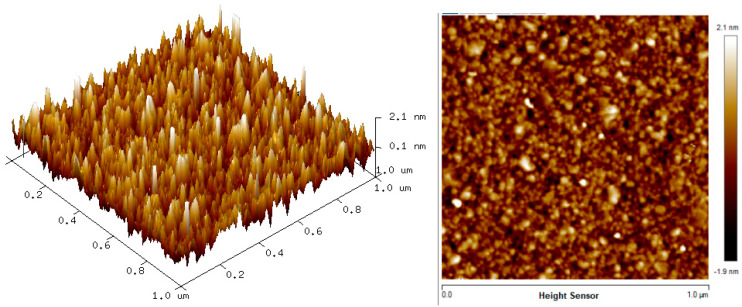
The AFM images of CTAB/HGQDs thin film after contact with the HMIs solution.

Furthermore, the AFM images of CTAB/HGQDs before and after coming in to contact with the metal ion solution were shown in Figure 13. After coming in to contact with the HMIs solution, the RMS roughness of CTAB/HGQDs thin film increased from 0.568 nm to 0.765 nm and a rougher surface was observed. This result is likely due to the interaction that happens between HMIs and the surface of the thin film, resulting in the increase of RMS roughness [80]. The AFM images also confirmed that the HMIs solution had already covered the surface of the thin film resulting in the changes of RMS roughness.

## 4. Conclusions

In this study, the CTAB/HGQDs thin film has been successfully prepared using the spin coating technique. The FTIR result confirmed that the existence of ammonium, carbonyl, carboxyl, and epoxy groups justified the interaction between CTAB and HGQDs. Additionally, AFM analysis revealed that the modification of HGQDs with CTAB smoothens the surface of the thin film with a homogenous surface. The absorbance value of CTAB/HGQDs was the highest compared to other thin films, at a wavelength of around 300 nm. Moreover, the PL of all thin films demonstrated emission at a wavelength of around 420 nm, which indicates the blue light in the visible light spectrum. The studies of CTAB/HGQDs thin film by using the SPR optical technique, successfully demonstrated that the proposed thin film can detect Zn^2+^, Ni^2+^, and Fe^3+^ by comparison with the SPR curve of deionized water. It can be concluded that CTAB/HGQDs have a high potential for HMIs detection using the SPR technique with the potential for further investigation. 

## Figures and Tables

**Table 1:** Comparison of SPR-based sensor for heavy metal ions (HMIs) detection.

Materials	HMIs	Detection Limit	References
metallothionein (MT)	Zn^2+^, Ni^2+^, Cd^2+^	~2 µM	[74]
polypyrrole-chitosan	Zn^2+^, Ni^2+^	Zn^2+^: 15 μM Ni^2+^: 17 μM	[75]
polypyrrole-multiwalled carbon nanotube	Fe^2+^	1.79 μM	[76]
deferoxamine self-assembled monolayer	Fe^3+^	2.1 µM	[77]
polypyrrole	Cu^2+^, Fe^3+^	~1.8 µM	[78]
citrate-capped silver nanoparticles as	Fe^3+^	13 mM	[79]
CTAB/HGQDs	Fe^3+^, Zn^2+^, Ni^2+^	~1.8 µM	This work
